# Correlation between the body balance and functional capacity from elderly with chronic vestibular disorders

**DOI:** 10.1590/S1808-86942011000600017

**Published:** 2015-10-19

**Authors:** Raquel Ferreira de Sousa, Juliana Maria Gazzola, Maurício Malavasi Ganança, Célia Aparecida Paulino

**Affiliations:** 1 Physical therapist; MSc at the Master's Body Balance Rehabilitation and Social Inclusion Program at the Universidade Bandeirante de São Paulo - UNIBAN, Brazil; 2 Physical Therapist, MSc and PhD in Sciences; Professor at the Body Balance Rehabilitation and Social Inclusion Program at the Universidade Bandeirante de São Paulo - UNIBAN, Brazil; 3 Otorhinolaryngologist; MSc and PhD in Sciences; Professor at the Body Balance Rehabilitation and Social Inclusion Program at the Universidade Bandeirante de São Paulo - UNIBAN, Brazil; 4 Pharmachologist; MSc and PhD in Experimental and Compared Pathology Professor at the Master's Body Balance Rehabilitation and Social Inclusion Program at the Universidade Bandeirante de São Paulo - UNIBAN, Brazil

**Keywords:** activities of daily living, health of the elderly, postural balance, vestibular diseases

## Abstract

**Abstract:**

Vestibular disorders are common among the elderly, mainly resulting in dizziness and imbalance - symptoms which can impact daily routine activities.

**Aim:**

To study the correlation between body balance and functional capacity and a comparison of risk of falls, actual falls and the functional capacity of the elderly with chronic vestibular dysfunctions.

**Materials and Methods:**

A cross-sectional, clinical and experimental study with 50 senior citizens - 60 to 86 years, with chronic peripheral vestibular dysfunction. These participants underwent body balance assessment by the Dynamic Gait Index (DGI) and functional capacity assessment by the Functional Independence Measure (FIM). The data was tested using the Spearman correlation and comparison tests, Mann-Whitney and Kruskal- Wallis, being α=5% (0.05).

**Results:**

There was a significant correlation between the total DGI score and all FIM scores, especially the total score (r=0.447; *p*<0.001) and loss of functional capacity in elderly patients with the highest risk of falling (*p*<0.001).

**Conclusion:**

There is a positive correlation between body balance and functional capacity in elderly patients with peripheral vestibular disorders, that is: the better the balance, the better the individual's functional capacity. In addition, a worse functional capacity increases the individual's risk of falling.

## INTRODUCTION

With the current growth in the incidence of chronic diseases, thanks to population aging, vestibular disorders have acquired a greater epidemiological relevance, considering that the dysfunctions and their multiple associated neurotological symptoms, such as vertigo, dizziness, body instability, hearing loss and tinnitus are directly associated with advanced age^1^.

The vestibular system contributes as a reference system, to which the other systems (visual and somatosensorial) may compare to in sensorial conflict situations. This system provides information to the Central Nervous System (CNS) on the orientation of the body in space, together with somatosensorial information. Thus, when the vestibular system is compromised with aging, such as, for example, through progressive degeneration and reduction in the number of labyrinthine hair cells and vestibular receptor ganglion cells, the CNS has difficulties in dealing with reduced or conflicting sensorial information^2^.

Moreover, many complaints are frequent among the elderly, and we highlight dizziness, vertigo and unbalance. In more than half the cases, balance disorders starts between 65 and 75 years of age, and in about 30% of the aged have this disorder at this age, which represents limiting factors in their lives^3^. This clinical finding is considered a geriatric syndrome of multifactorial causes, which happens in multiple system deficits, causing the elderly major vulnerability facing daily challenges^4^.

By 65 years of age, dizziness is considered the second most prevalent symptom worldwide; after 75 years of age it becomes the number one symptom in prevalence, reaching 80% of the individuals in this category^1^.

One of the most disabling symptom of dizziness and body balance disorder is falls. These are defined as a non-intentional event resulting from the individual's change of position to a lower level in relation to the initial position, without an intrinsic determining factor such as a syncope, stroke or unavoidable accident^5^.

It is important to consider that most of the elderly develop along their lives some chronic disease caused by the continuous loss of function in organs or biological systems. This loss may or may not impair functional capacity which, in its turn, may trigger functional limitation and, ultimately, cause the elderly disabilities in doing the essential activities of daily life^6^.

A household survey with the elderly population of the city of São Paulo (SP), reported that only 53% of the individuals assessed had total autonomy to perform their daily activities, such as: prepare meals, clean the house, take their medication at the right time, comb one's hair, walk on a flat surface, eat, shower, among others. Moreover, 29% required partial or full help to finish up to three activities and 10% required help in more than three of the activities mentioned. These results suggest that longevity may no longer be an achievement, and it may become a nuisance as long as the increase in life expectancy is not matched by good health, autonomy and independence to perform daily activities^7^.

A cross-sectional study assessed the importance of motor performance in maintaining one's independence concerning daily activities such as travelling, shopping, preparing meals and cleaning the house. Results have shown that there is a strong association between less balance and a loss in the execution of instrumental activities by the elderly in the community^8^. Nonetheless, there were no explanations as to the degree of correlation between balance and the functional capacities of senior citizens with vestibular diseases, assessed by the Functional Independence Measure (FIM), in regards of basic daily-life activities, such as dressing, showering, undergoing postural transfers, and others.

Having said that, this study aimed at investigating the correlation between body balance and functional capacity in the elderly with chronic peripheral vestibular disorders and to compare the risk of falls and the occurence of falls with the functional capacity of these individuals.

## MATERIALS AND METHODS

We carried out a cross-sectional, descriptive and analytical study with 50 senior citizens (N = 50), between 60 and 86 years of age, from both genders, with peripheral vestibular dysfunctions.

Inclusion criteria for participation were: elderly individuals with 60 years or more of age; men and women; with vestibular disorders, dizziness and reduction in body balance and stunning or a feeling of unspecific dizziness, with peripheral etiology and daily occurence, - weekly, monthly or sporadic, for at least three months.

From this study, we took off those individuals who had sensorial, cognitive or physical disabilities, which prevented the execution of body balance assessment, as well as having incapacity to understand simple verbal commands and questions associated to FIM.

We also took off the elderly with vertigo spells; patients with severe vision and hearing impairment - considered incapable to perform their daily activities, even with corrective lenses or hearing aids; elderly with amputation of the upper and/or lower limbs; patients unfit for walking independently, and those in a body balance rehabilitation program for at least six months before this study was carried out.

This study was previously approved by the Ethics in Research Committee (Protocol # 032/2009) and only the elderly who signed the Informed Consent Form were enrolled in the study.

The elderly with a diagnostic hypothesis of chronic peripheral vestibular dysfunction were submitted to assessment by an ENT physician and, through clinical assessment and complementary tests, they were diagnosed with vestibular dysfunction. The clinical assessment, the tests and the clinical diagnosis were recorded in medical charts, which were later assessed based on the criteria of eligibility; and those elderly who fit the study's inclusion criteria were pre-selected.

The pre-selected elderly were promptly instructed and invited to participate in the study. After proper acceptance by signing the Informed Consent Form, they were submitted to physical therapist evaluation by means of the Functional Assessment Form, the *Dynamic Gait Index* (DGI)^9^ and the FIM^10^, between September of 2009 and September of 2010.

The functional assessment tool was made of a series of questions associated with aging: gender; age; number of medications used, type of peripheral vestibular dysfunction diagnosed; main symptoms; dizziness characterized as to type, duration, frequency and type of rotational dizziness, and falls in the past six months.

In order to assess body balance, we selected the DGI - a functional scale made up by eight tasks involving gait in different sensorial contexts, including flat surface, changes in gait speed, horizontal and vertical movements with one's head, to pass over and go around obstacles; turn on one's own body axis; climb up and down stairs. The maximum score was 24 points and a score of 19 points or less is predictable of gait instability and a higher risk of falls^11^.

We used the FIM in order to measure functional capacity, for it is a reliable scale, with international validity - which has been validated and reproduced nationally. This scale is made up of six dimensions, of which four are motor: self-care, sphincter control, transfers and locomotion; and two are cognitive: social cognition and communication. For the purposes of the present study, we used the score resulting from the motor and cognitive aspects of the FIM and the summation of these dimensions^10^.

The patient was submitted to assessment by the FIM and, during the questions associated with motor issues, each item answered was checked, asking the patient to show each task, so that the scores would be defined according to the individual's true capacity.

We carried out a simple descriptive analysis and we applied the Spearman Linear Correlation, the Mann-Whitney and Kruskal-Wallis tests, with an alpha level of 0.05 (5%).

## RESULTS

The study assessed 50 elderly (N = 50) with ages varying between 60 and 86 years, with mean age equal to 69 years; median equal to 68 years and standard deviation (SD) equal to 6.7. The percentage distribution of the age ranges were: from 60 to 69 years (66.0%), between 70 and 79 years (22.0%) and from 80 to 89 years (12.0%) and, as far as gender is concerned, there was a predominance of women, corresponding to 40 elderly women (80.0%).

In relation to the number of drugs taken, 45 elderly (90%) used some medication. Of these, 15 individuals (30.0%) used five or more. As to vestibular disorders, Benign Positional Paroxysmal Vertigo (BPPV) was the most frequent vestibular disorder, affecting 14 elderly (28%). As to the results from the caloric test, 35 elderly had normal reflexes, corresponding to 82% of the sample. As to the dizziness-triggering symptoms, body instability was the most frequent symptom in the sample, being found in 39 elderly (78%).

About the dizziness-triggering positions, we noticed that keeping the head in a specific position was the most frequent cause, being observed in 30 individuals (60%).

Rolling one's head to the right, or to the left, was the movement that most triggered dizziness, according to the report from 26 elderly (52 %).

As to falls, 33 elderly did not fall (74% of the sample), 13 individuals fell (26% of the sample); of whom, 18% (nine individuals) had one fall and 8% (four individuals had had at least two falls in the past six months.

Dizziness was seen in 100% of the elderly with chronic peripheral vestibular diseases. The predominant dizziness onset time was five years, or more, seen in 54% of the sample. In relation to the other characteristics of dizziness, we stress the following sample percentages: type of rotation dizziness (42%), type of objective and subjective rotational dizziness (26%), duration of seconds (44%) and of sporadic frequency (66%).

In relation to the elderly body balance, assessed by means of the total DGI score, we found a mean of 18.5 points and a median equal to 19 points, varying between 9 and 24 points; and a SD = 3.01. According to the total DGI score, we classified the risk of falls from each participant, and the score between 0 and 19 corresponded to a greater risk of falls; a score between 20 and 24 was associated to a lower risk of falls. We also noticed that from the 50 elderly assessed, 31 participants (62%) had a greater risk of falling (*p* = 0.016).

In regards of the functional capacity of the elderly in the study, assessed by the total FIM score, we found a mean value of 118.5 points and median equal to 120 points, with a variation between 103 and 126 points; and SD = 5.9. Considering the FIM total score classification, we stress the modified independence category (111 - 125 points), seen in 42 elderly (84%); total independence category (126 points) and mild dependence (103 - 107), corresponding to 10% and 6%, respectively.

In relation to the correlation test between body balance and functional capacity, we noticed a statistically significant correlation between the total DGI score and all FIM domains, stressing the correlation between DGI and total FIM (r = 0.447; *p* = 0.001), as per depicted on Table 1.

**Table 1:** Analysis of the correlation between motor FIM, cognitive FIM, Total FIM and Total DGI of 50 elderly with chronic peripheral vestibular dysfunction.

Variables	R	%	Spearman's Correlation (*p*-value)
DGI x Motor FIM	0,364	36,40	0,009
DGI x Cognitive FIM	0,282	28,20	0,047
DGI x Total FIM	0,447	44,70	0,001

DGI: Dynamic Gait Index.

FIM: Functional Independence Measure.

Comparing the risk of falls with the functional capacity, we noticed that there was a statistically significant difference between the DGI categories for all FIM domains, in other words: motor FIM x categorical DGI (*p*<0.020); Cognitive FIM x categorical DGI (*p*<0.015), and total FIM x categorical DGI (*p*<0.001). Considering the total FIM domain, the elderly with the greater risk of falls had lower scores in the total FIM domain score, as per shown on Table 2.

**Table 2:** Comparative analysis of the average results of the Functional Independence Measure (total, motor and cognitive) for a higher and lower risk of falls among 50 elderly with chronic peripheral vestibular dysfunction.

FIM	Greater risk	SD	Lower Risk	SD	Mann-Whitney (*p*-valor)
Total	116,5	5,9	121,8	4,2	0,001
Motor	86,0	4,2	88,8	2,7	0,020
Cognitive	30,5	3,5	33,0	2,2	0,015

In relation to comparing fall occurrence categories with functional capacity, we noticed that there were no statistically significant differences among the fall occurrence category for all FIM domains, as per shown on Table 3.

**Table 3:** Comparative analysis of the average results of the Functional Independence Measure (total, motor and cognitive) for no falls, one fall and two falls in over 50 elderly with chronic peripheral vestibular dysfunctions.

Functional Independence Measure	None	One fall	Two or more falls	Kruskal Wallis (*p*-value)
Total	118,6	119,9	114,5	0,195
Motor	87,2	87,6	85,0	0,299
Cognitive	31,4	32,3	29,5	0,241

## DISCUSSION

In relation to the age range of the elderly who participated in the study, we computed a mean age of 69 years (66% of the sample), corroborating a previous study, which reported that in an elderly population they found 58% of the individuals below 70 years of age and a mean age of 69 years^7^. In another study, researchers also found a greater prevalence of elderly with peripheral vestibular dysfunctions in the age range between 60 and 69 years^12^.

The predominance of elderly in this age range can be explained by the fact that, during this period, most elderly individuals are still active and take good care of themselves, looking for medical care more often and, consequently, increasing the number of elderly with vestibular disorders in this age range. Moreover, mortality rates grows with age, thus reducing the number of elderly diagnosed with vestibular dysfunction as age advances^13^.

In this study, most participants were women (80%), which is in agreement with reports found in the literature^14^, ^15^, ^16^. Dizziness is more frequently found among women, at the rate of 2:1. This ratio, according to some authors, can be justified by the association of vestibular disorders with hormonal and metabolic changes, besides the fact that women are often more concerned about seeking medical care and guidance when compared to men^17^.

In the current study, most of the elderly (90%) used some kind of medication; these patients - users of five or more medications - corresponded to 30% and the mean value found was 2.9 drugs per patient, similar to what happened to the elderly assessed in a previous study^18^. This can be justified by the greater health compromise in elderly with vestibular diseases, who need a higher number of medication.

It is important to stress that the elderly with chronic vestibular dysfunction of this study had an average of almost three drugs taken together and poly-pharmacotherapy, besides favoring an increase in the risk of drug interactions and adverse events, it may worsen labyrinthine symptoms, and it may impair body balance^1^.

Among vestibular syndrome etiologies, BPPV was the most prevalent, happening to 28% of the elderly assessed. This result is also in agreement with previous studies^19^,^20^, given that these scholars also reported BPPV as the most frequent vestibular disorder among peripheral vestibular diseases, especially among the elderly.

This disease is also the most frequent in women, at a ratio of 2:1 in the group with idiopathic BPPV, and of 1:1 when the etiology is defined^21^. The higher frequency in women may have contributed to a predominance of BPPV, among the peripheral vestibular diseases investigated in this study, having seen that the elderly women represented 80% of the sample evaluated.

We noticed that the caloric test – study of the post-caloric nystagmus – showed that most elderly had normoreflexia as the result, as it has been already reported^18^. Such finding was, very likely observed because of this test's low sensitivity to detect peripheral vestibular dysfunctions. In another study^22^, we noticed a sensitivity around 50% to detect changes in the caloric test in individuals with peripheral vestibular dysfunction, stressing the importance of assessing the patient's clinical history and the detailed clinical study of the signs and symptoms for a proper clinical diagnosis. In this study we did not notice pathognomonic signs of central disorders upon the vestibular exam, due to the exclusion of elderly individuals with central vestibular disorders.

The most reported triggering movements and positioning in this study were: to keep the head in a specific position, to turn the head, to stand up from laying down and walk - similar to what we already find in the literature^4^, which most mentioned positions and activates were to stand up from laying down, to turn the head, to turn the body and stand up from laying down.

Is it possible that these head movements and/or position be very stimulating to the vestibular system, and may cause vertigo and other dizziness, as in the BPPV cases seen in clinical practice.

About the prevalence of the associated symptoms in the elderly enrolled in the present study, the results are in agreement with papers already published^18^,^22^, which found an association of dizziness with tinnitus (79.4%), hipoacusia (55.9%), sensitivity to intense sounds (47.1%), neurovegetative disorders (55.9%) and syncope in 8.8% of the cases, also in senior citizens with vestibular disorders. These findings corroborate the fact that elderly individuals with vestibular disorders tend to have a concurrent involvement of other systems associated with vestibular function, the auditory among them.

In the present study, concerning the occurrence of falls, there was a predominance of elderly who did not fall, that is, who did not have fall episodes in the past six months, making up 74% of the sample; followed by individuals with only one episode of fall (18%) and others with two falls or more (8%). These results may be justified by the fact that these elderly presented a functional interdependence profile adapted to the limitations of the balance disorder stemming from the vestibular dysfunction, in other words, even facing the body instability and the greater risk of falls, triggered by the behavior of sensorial systems associated with postural control, these individuals may have acquired motor adaptations in order to avoid a fall during the DLA, such as reduction in gait velocity, increasing the support basis; less exposure to extrinsic factors associated with falls; greater dependence on visual clues, and restriction of activities bearing a greater risk o falls.

In regards to the elderly who fell (26% of the sample), it was not possible to find out whether the fall reported was accidental or if it was associated with a vestibular dysfunction or a disorder of body balance, given that the causal factor was not investigated in this population. Therefore, the falls reported may have happened due to intrinsic factors which were not associated with the vestibular dysfunction, but rather to the very age-related physiological changes, such as visual disorders; reduction in muscular strength, reduction in the proprioception sensitivity of the lower limbs, or even by extrinsic accidental factors such as a barrier on the ground; slippery surfaces; sudden breaking of a collective means of transportation; the use of stairs, etc., not worsening the functional capacity of these elderly.

As to the characterization of the dizziness spells, 100% of the patients reported they had dizziness, and 54% said they had had dizziness for five years or more. Rotational dizziness was the most prevalent (42%), being objective and subjective (26%), lasting for seconds (44%) and being sporadic in 66% of the elderly. The data we obtained associated with dizziness are in agreement with the results from the study of clinical characterization of elderly with chronic vestibular disorders^16^, which reported that 43.3% of the cases had dizziness for over five years.

However, this study disagrees with other results reported by a previous study^16^ as to the type, duration and periodicity; these authors found a predominance of the rotational and non-rotational dizziness, lasting for minutes and repeating daily. Such results reveal characteristics of a more frequent, persistent and varied dizziness, commonly seen in patients with more severe cases and those with vestibular dysfunction disabilities. In the present study, the elderly who participated in it had milder characteristics of dizziness, very likely because they were better adapted, more active, independent people (from the functional stand point) and some of them had milder chronic peripheral vestibular dysfunction.

In the present study, the mean DGI score was 18.5, in disagreement with studies by Whitney et al.^23^, who detected a mean score of 21, and from Cohen & Kimball^24^, who found a mean score of 21.4 in patients with vestibular disorders, with mean age of 61 and 57.4 years, respectively. This lower DGI mean found in the present study is very likely justified by the higher mean age of the elderly assessed (69 years).

Still, according to the total DGI score, classified the risk of falls from each participant, and the score from 0 to 19 corresponded to a higher risk of falls; the score between 20 and 24 was associated with a lower risk of falling. Thirty-one elderly (62%) had a greater risk of falling. A reduction in falling has been associated with a higher risk of falls^25^. Also, the increase in body oscillation is directly related to an increase in the frequency of falls^21^.

The predominance of individuals with a higher risk of falls (62%) in the present study can be explained by the fact that the elderly with chronic peripheral vestibular dysfunctions and body balance disorder have an important loss in their sensorial systems, especially visual and vestibular, producing inadequate motor responses and body mass center projection close to the body's limit of stability, resulting in a greater risk of falling^26^.

Regarding functional capacity, in the present study, the mean FIM score was 118.5 points and the median was 120 points, in other words, most of the elderly were considered self-sufficient to perform their functional activities. This study can be justified by the very profile of the elderly who made up the study sample, in other words: individuals with mild dizziness symptoms, relatively young, some still very active and practicing regular and adapted physical activities, and also participants of social activities promoted by the community. Nevertheless, these elderly did not reach the total score, because although not needing the help of others to perform their functional activities, these individuals had a slow gait; reduction in the head movement speed during gait, and lack of self-assurance to perform the activity for fear of triggering dizziness - even if sporadic.

Such finding was in disagreement from those reported by Ramos et al.^7^, showing that about 40% of the individuals with 65 years or more require some type of help to perform at least one task, such as shopping, taking care of financial issues, preparing meals and cleaning the house. A smaller share (10%), nonetheless significant, requires help to perform basic tasks such as showering, dressing, using the toilet, sitting down and standing up from chairs and beds.

On the other hand, marked dependence and associated with a high degree of functional disability is not a universal fact in old age^27^. Epidemiological studies have shown that only 4% of the elderly with more than 65 years of age had marked disability and a high degree of dependence; besides the 13% of those between 65 and 74 years, and 25% of those in the age range above 85 years.

In the present study, the correlation test between body balance and functional capacity revealed a statistically positive correlation between the total DGI score and the total FIM score. This result shows that the independence towards gait and balance are inherent factors to a good functional capacity^28^.

The positive correlation between body balance and functional capacity can be justified by the fact that the vestibular dysfunction makes it difficult to see and perceive position and movement; it impacts the body's vertical orientation, vision and head stabilization, and it impairs body mass center control, causing unbalance, characterized by an increase in body oscillation, reduction in the stability limit, individual's performance reduction upon gait and increase in the risk of falls. Consequently, this individual with body instability reduces his active mobility; is afraid of falling; loses physical conditioning; is afraid of falling, and reduces social participation and motor skills to perform functional activities indepedently^11^.

Moreover, this correlation may be explained by the fact that the gait-depending tasks seem to become more difficult in the elderly with chronic vestibular dysfunction, as the environment requires greater postural control. Thus, to go up and down stairs, go shopping and leave a bus or a car may be more challenging tasks than cleaning the house, for instance, since the stabilization (head and visual), head and trunk movements and, especially, body balance, to face obstacles which may eventually appear, are constantly required in the external environment^29^.

The same problem has been reported, adding that gait with the horizontal head movement was the most difficult to perform when compared to the head's vertical movement^30^. DGI tasks, which the head is in movement, in horizontal or vertical rotation are the most difficult to be performed and are more related to functional disability and falls^31^.

With this study, we can see that the physiological decline observed during the aging process, associated to the onset of chronic peripheral vestibular dysfunction, may contribute together to the loss in body balance and functional capacity; however, it is important to stress the need to carry out new studies comparing the relationship of these parameters in the experimental and control groups, in other words, in the elderly with chronic peripheral vestibular dysfunctions and healthy elderly.

Concerning the risk of falls and functional capacity, senior citizens with a greater risk have a reduction in such capacity, having seen that the vestibular dysfunction they have brings about body balance disorders, such as a difficulty in controlling and guiding head and body posture in the vertical plane; difficulty in controlling the body mass center within the support basis and, especially, difficulty in walking and simultaneously moving the head horizontally and vertically.

We did not find significant differences between the response levels of “Fall in the past 6 months” variable for FIM, and it may be due to the fact that most of the elderly interviewed did not fall (74% of the sample). As per discussed before, the elderly in the present study had a functional independence profile adapted to the lack of body balance situation which is characteristic of their vestibular dysfunction, in other words, even facing body instability and a greater risk of falls, triggered by the involvement of sensorial, vestibular, visual and somatosensorial systems, these individuals must have acquired motor adaptations in order to avoid falling during Daily Life Activities (DLA).

Thus, we observed a greater trend towards falls because of the body balance disorder condition triggered by the vestibular dysfunction. However, in the sample considered, body instability with an increase in the risk of falls, was not enough to cause many fall events, very likely because of the motor adaptations acquired by the elderly, which had good functional capacity indices, as per shown in the present study.

Still, regarding the elderly who fell (26% of the sample), the reported episodes of falls may have happened because of the body balance involvement caused by the vestibular dysfunction, focusing on the importance of body balance rehabilitation in these elderly towards the prevention of falls and disabling secondary complications.

These falls may also have happened accidentally – not being associated with the vestibular dysfunction and body balance, which were not enough to cause a significant decline in functional capacity concerning motor and cognitive activities, and their specific association in the sample investigated.

Since body balance is directly associated with the functional capacity, intervention programs bearing exercises capable of helping maintain such balance, may favor maintenance and even an improvement in functional capacity, providing greater independence in the performance of DLA and, consequently, better quality of life and social inclusion for the elderly with vestibular disorders.

## CONCLUSION

We have concluded that the better the body balance, the better is the functional capacity of senior citizens with chronic peripheral vestibular dysfunctions and, still, the greater the loss in functional capacity, the higher is the risk of these individuals suffering falls.
